# Inhalation Toxicity of Brevetoxin 3 in Rats Exposed for Twenty-Two Days

**DOI:** 10.1289/ehp.7497

**Published:** 2005-02-09

**Authors:** Janet M. Benson, Fletcher F. Hahn, Thomas H. March, Jacob D. McDonald, Andrea P. Gomez, Mohan J. Sopori, Andrea J. Bourdelais, Jerome Naar, Julia Zaias, Gregory D. Bossart, Daniel G. Baden

**Affiliations:** ^1^Lovelace Respiratory Research Institute, Albuquerque, New Mexico, USA;; ^2^Center for Marine Science Research, University of North Carolina at Wilmington, Wilmington, North Carolina, USA;; ^3^Department of Pathology, University of Miami, Miami, Florida, USA;; ^4^Division of Marine Mammal Research and Conservation, Harbor Branch Oceanographic Institution, Ft. Pierce, Florida, USA

**Keywords:** brevetoxin, immunotoxicity, inhalation, neurotoxicity, rats

## Abstract

Brevetoxins are potent neurotoxins produced by the marine dinoflagellate *Karenia brevis*. Exposure to brevetoxins may occur during a *K. brevis* red tide when the compounds become aerosolized by wind and surf. This study assessed possible adverse health effects associated with inhalation exposure to brevetoxin 3, one of the major brevetoxins produced by *K. brevis* and present in aerosols collected along beaches affected by red tide. Male F344 rats were exposed to brevetoxin 3 at 0, 37, and 237 μg/m^3^ by nose-only inhalation 2 hr/day, 5 days/week for up to 22 exposure days. Estimated deposited brevetoxin 3 doses were 0.9 and 5.8 μg/kg/day for the low-and high-dose groups, respectively. Body weights of the high-dose group were significantly below control values. There were no clinical signs of toxicity. Terminal body weights of both low- and high-dose-group rats were significantly below control values. Minimal alveolar macrophage hyperplasia was observed in three of six and six of six of the low- and high-dose groups, respectively. No histopathologic lesions were observed in the nose, brain, liver, or bone marrow of any group. Reticulocyte numbers in whole blood were significantly increased in the high-dose group, and mean corpuscular volume showed a significant decreasing trend with increasing exposure concentration. Humoral-mediated immunity was suppressed in brevetoxin-exposed rats as indicated by significant reduction in splenic plaque-forming cells in both low- and high-dose-group rats compared with controls. Results indicate that the immune system is the primary target for toxicity in rats after repeated inhalation exposure to relatively high concentrations of brevetoxins.

Blooms of the dinoflagellate *Karenia brevis* are responsible for what are commonly referred to as Florida red tides. *K. brevis* produces a series of potent neurotoxins known as brevetoxins (Baden 1989). The reader is referred to reviews by Fleming et al. (2005a) and Kirkpatrick et al. (2004) for discussion of Florida red tide exposures, effects, and implications for human health.

*K. brevis* red tides occur almost annually in the Gulf of Mexico and have increased in geographic distribution since the 1970s (Van Dolah 2000). Therefore, the possibility of repeated inhalation exposure for individuals working and living along affected beaches and waterways is increasing. Despite this, little is known about the possible systemic health effects associated with aerosolized brevetoxins beyond the obvious immediate upper respiratory tract irritation (Backer et al. 2003; Kirkpatrick et al. 2004). Bossart et al. (1998) reported respiratory tract inflammation and hemopathy in manatees dying during extensive 1996 *K. brevis* red tides. Immunohistochemical staining of tissues from these manatees indicated an accumulation of brevetoxins in tissue macrophages and lymphocytes, key players in humoral and cell-mediated immune responses. Although these effects in manatees occurred after weeks of exposure via several routes, the data suggest that the respiratory tract, hematopoietic, and immune systems might be targets for brevetoxin-induced toxicity after repeated exposure of humans to environmentally relevant airborne concentrations of brevetoxins.

In animal studies with inhaled brevetoxins, suppressed splenic antibody production was observed among Sprague-Dawley rats inhaling aerosols of crude *K. brevis* extract 4 hr/day for 1 and 4 weeks. No toxicity to the nervous, respiratory, or hematopoietic systems was noted (Benson et al. 2003). The extract contained primarily brevetoxins 2 and 3 but also contained brevenal, a newly identified compound in *K. brevis* having pharmacologic activity antagonistic to brevetoxin-induced neurotoxicity and bronchoconstrictor activities (Bourdelais et al. 2003; Abraham et al. 2005). Brevetoxin-induced suppression of splenic antibody production was confirmed in rats inhaling pure brevetoxin 3 at 500 μg/m^3^ for 0.5 hr and 2 hr/day for 5 consecutive days (Benson et al. 2004). Antibody production was suppressed by > 70% in the low-exposure (0.5-hr exposure/day) and high-exposure (2-hr exposure/day) groups. Small numbers of splenic and peribronchiolar lymphoid tissue macrophages stained positive for brevetoxin. No biochemical or histologic evidence of toxicity to the respiratory, nervous, or hematopoietic systems was found in the rats inhaling pure brevetoxin 3 for 5 days.

The purpose of the study reported here was to extend our investigation of the adverse systemic health effects associated with brevetoxin inhalation exposure, exclusive of acute respiratory tract irritation. Brevetoxin 3 was chosen for these studies because it is a major component of the brevetoxin mixture produced by *K. brevis* (Baden 1989) and of brevetoxin-containing aerosols measured along red tide affected beaches (Cheng et al. 2005). The exposure scenario was chosen to more closely mimic a 5 day/week occupational exposure than occasional recreational exposure, although daily exposure durations were much shorter (2 hr) than those expected for occupational exposures. The aerosol concentrations employed were 2–3 orders of magnitude higher than total brevetoxin concentrations measured to date along Florida beaches during red tides of mild to moderate intensity (Backer et al. 2003; Cheng et al. 2005; Pierce et al. 2003).

## Materials and Methods

### Chemicals

Brevetoxin 3 was isolated and purified from the Wilson clone of *K. brevis* at the Center for Marine Sciences, University of North Carolina, Wilmington. Unless otherwise specified, all other chemicals, including ^3^H-thymidine-5 (20–30 Ci/mmol; 95% radiochemically pure), were purchased from Sigma Chemical Company (St. Louis, MO).

### Animals

Male F344/CrlBr rats, 6–7 weeks old when received from Charles River Laboratories (Wilmington, MA), were used. The rats were housed in polycarbonate cages with hardwood chip bedding. The animal rooms were maintained at 20–22°C with relative humidity at 20–50% and a 12-hr light cycle beginning at 0600 hr. Food [Harlan Teklad rodent diet (W), Madison, WI] and water were provided ad libitum. The rats were randomized by weight into exposure groups and weighed 230.3 ± 8.3 g (mean ± SD; *n* = 66) when exposures began. The rats were conditioned to the nose-only inhalation restraint tubes for 0.5, 1, and 2 hr before initiation of exposures. The study protocol was reviewed and approved by the Lovelace Respiratory Research Institute Institutional Animal Care and Use Committee.

### Exposure System

The exposure system consisted of three, 36-port cylindrical nose-only inhalation chambers (InTox Products, Edgewood, NM), each supplied with a single nebulizer (Hospitak, Inc., Farmingdale, NY). Aerosols generated in the nebulizer were dried and diluted with supply air to achieve the desired chamber aerosol concentrations. Flow rate through the chamber was 10 L/min. Temperatures were monitored continuously with an acceptable range of 18–22°C. Chamber oxygen concentration was monitored continuously with an action level at ≤18%.

### Aerosol Generation and Characterization

The aerosols used in this study were saline based, to mimic sodium chloride–based marine aerosols. Stock solutions containing 0.5 mg brevetoxin/mL were prepared in 100% ethanol. Generator solutions for the nebulizers supplying the low- and high-level exposure chambers were prepared daily by diluting the stock solutions with 0.9% saline to achieve a final concentration of 0.15 mg/mL. The generator solution for the control chamber was ethanol–saline. Target total exposure concentrations were 5 mg total aerosol/m^3^ for the low-level exposure chamber and 20 mg total aerosol/m^3^ for the control and high-level exposure chambers. Target exposure concentrations were achieved by operating the nebulizer at 2 psi for the low-level exposure chamber and 4 psi for the control and high-level exposure chambers. The total aerosol concentrations of the saline-based aerosols in the exposure atmospheres were determined gravimetrically. Aerosol was collected from the breathing zone of the animals at a flow rate of 2 L/min onto preweighed 25-mm Zefluor filters (SKC Gulf Coast, Houston, TX) at 0.5-hr intervals. The stability of the total aerosol concentration during the exposure was monitored using a TSI DustTrak real-time aerosol monitor (TSI Industries, Shoreview, MN). The particle size distributions [mass median aerodynamic diameter (MMAD) and geometric standard deviation (σ_g_)] of the aerosols were determined using an eight-stage cascade impactor (InTox Products).

To directly quantitate brevetoxin concentration in the exposure atmosphere, selected filter samples were extracted with acetone (Fisher Scientific, Fairlawn, NJ), dried, and resuspended in methanol and water (50:50). Extracts were spiked with brevetoxin 2 as an internal standard. Samples were injected onto a high-pressure liquid chromatograph (10ADVP; Shimadzu Company, Kyoto, Japan) equipped with a 75 × 2-mm analytical column (Aqua 3 μm, Phenomenex USA, Torrance, CA). Brevetoxin was eluted using a methanol–water mobile phase containing 1 mM ammonium acetate. Eluant was directed into an electrospray mass spectrometer (API 365; Applied Biosystems, Foster City, CA). The mass spectrometer was monitored for ion pairs consisting of 897.590/725.404 (brevetoxin 3) and 895.559/877.550 (brevetoxin 2). Aerosol concentrations were confirmed by enzyme-linked immunosorbent assay (ELISA) analysis (Naar et al. 2002) performed on the above filter extracts.

To determine the concentration of ethanol vapor in the exposure atmosphere, a sample of the control chamber exposure atmosphere was collected into a Tedlar bag obtained using an SKC Vac-U Chamber (SKC Inc., Eighty Four, PA). A glass-fiber filter was placed in line to remove particles from the sample before it entered the sampling bag. The ethanol concentration in the sample was measured by gas chromatography with flame ionization using a Shimadzu Model GC-17A/FID (Shimadzu Scientific Instruments, Columbia, MD) equipped with a Restek Rtx-1 column (30-m × 0.32-mm × 5-μm film width; Restek Corporation, Bellefonte, PA). The chromatograph oven was operated isothermally at 50°C. The injector and detector temperatures were 200°C. A five-point ethanol vapor standard curve was over a concentration range of 1.16–9.4 g/m^3^ was used.

### Experimental Design

Subgroup A rats (*n* = 6 per exposure level) were exposed 2 hr/day, 5 days/week for a total of 22 exposure days. End points specific to this group included gross and histopathology, hematology, serum chemistry, and evaluations of bronchoalveolar lavage fluid for indications of cytotoxicity [lactate dehydrogenase (LDH) activity] and inflammation (total protein, total and differential nucleated cell counts), and splenocyte proliferation in response to mitogen *in vitro*. Subgroup B rats (*n* = 10 per exposure level) were sacrificed after 5 and 22 days of exposure (*n* = 5 per sacrifice time) for quantitation of brevetoxin 3 or metabolites in liver, the organ expected to have the greatest dose (Benson et al. 1999; Cattet and Geraci 1993; Poli et al. 1990), and for evaluation of splenocyte antigen recognition *in vitro* after 22 days of exposure.

### *In Vivo* Observations

All rats were observed for clinical signs of toxicity, especially neurotoxicity, after each day’s exposure. In addition, the rats were weighed, and detailed clinical observations were recorded weekly (Path Tox Software; Xybion, Cedar Knolls, NJ).

### Necropsy

All rats were sacrificed by intraperitoneal injection of an overdose of Euthasol (Virbac AH Inc., Fort Worth, TX). Body weights were recorded for all rats at the 4-week (terminal) sacrifice. Blood was collected from subgroup A rats by cardiac puncture for evaluation of hematology and serum chemistry. Each subgroup A rat (*n* = 6) received a complete necropsy. Brain, lung, liver, kidney, and spleen weights were recorded. To obtain bronchoalveolar lavage fluid, the left lung bronchus was clamped, and the right lobes were lavaged twice with 4 mL physiologic saline. After the lavage, the right bronchus was clamped, and the left bronchus was unclamped. The left lobe was fixed via the trachea using 10% neutral-buffered formalin. The nose, brain, liver, kidney, femur with marrow, and spleen (half) were also fixed in 10% neutral-buffered formalin for histologic evaluation. Splenocytes were isolated from the remaining half of spleen for evaluation of their proliferative response to mitogen challenge *in vitro*.

### Histopathology

The soft tissues were trimmed and embedded in paraffin. Tissues were sectioned at 5 μm and stained with hematoxylin and eosin. To more closely examine brain tissue for neuronal damage, three cross sections of brain (level of optic chiasma, caudal to mammillary bodies and caudal to transverse fibers of the pons) were stained with luxol fast blue-cresyl violet. These cross-sections contain profiles of the cerebellar cortex, hippocampus, and thalamus. The focus was on the neurons in these regions because of indications of neuronal damage and loss in these regions in mice inhaling brevetoxin 3 (Murray TF, unpublished results) and because of the defined nature of these groups of neurons.

### Clinical Pathology

Hematologic evaluations on whole blood obtained at necropsy were performed using an Advia 120 Hematology System (Bayer, Terrytown, NJ). Clinical chemistry evaluations on serum were performed using a Hitachi 911 Automatic Analyzer (Roche Diagnostic Corp., Indianapolis, IN). Standards and reagents were purchased from Pointe Scientific (Lincoln Park, MI, and Diagnostic Chemical Ltd., Oxford, CT).

### Lavage Fluid Analysis

The volume of fluid recovered in the first lavage wash was recorded. Nucleated cells were sedimented by centrifugation. The supernatant from the first lavage was analyzed for LDH and total protein using the Hitachi 911 Automatic Analyzer. The cell pellets from both washes from each animal were pooled and counted manually using a hemocytometer. Differential counts of nucleated cells were made on cytocentrifuge preparations stained with Kwik Diff (Shandon, Inc., Pittsburgh, PA).

The state of activation of the macrophages recovered in lavage fluid was determined using a zymosan-stimulated chemiluminescence assay (Benson et al. 2004; Hubbs et al. 2001). The assay was conducted on 2 × 10^5^ alveolar macrophages per sample well in a total volume of 0.15 mL 1-piperazine ethane sulfonic acid, 4-(2-hydroxyethyl)-monosodium salt (HEPES) buffer.

### Brevetoxin Concentration in Liver

Rats were sacrificed by intraperitoneal injection of Euthasol approximately 24 hr after exposure. The liver was removed, weighed, and frozen (−20°C) pending analysis. Thawed tissues were homogenized and 1-g aliquots of tissue homogenate were extracted three times with 4 mL acetone. The acetone extracts for each sample were analyzed for brevetoxin 3 or metabolites by ELISA as described by Naar et al. (2002).

### Immune Responses

#### Antibody-forming cell response.

The immunoglobin M (IgM) antibody-forming cell response to the T-cell–dependent antigen sheep red blood cells (SRBCs) was assessed with a modified plaque-forming assay (Cunningham and Szenberg 1968). Subgroup B rats were immunized by tail vein injection (250 μL of a 15% suspension of SRBC in phosphate buffered saline). At 7 days postimmunization the rats were euthanized by intraperitoneal injection of an overdose of Euthasol. Cells were isolated from weighed portions of spleen, washed, and then resuspended in complete RPMI culture medium to a final concentration of 1 × 10^6^ cells/mL. Analyses were performed as described previously (Benson et al. 2004).

#### Spleen lymphocyte proliferation.

The proliferative response of the spleen cells to the mitogen, concanavalin A (Con A) was assessed. Group A spleen cells (5 × 10^5^/100 L complete RPMI medium) were incubated with 0.1, 0.3, and 1.0 μg Con A/50 μL, respectively, at 37°C in the presence of 5% CO_2_ for 54 hr. At that time, cells were pulsed with 0.5 μCi ^3^H-thymidine and incubated for an additional 18 hr. Cells were collected on filter paper using a cell harvester, and ^3^H activity (decays per minute) was determined by liquid scintillation spectrometry.

### Statistical Analyses

Means, standard deviations, and standard errors for experimental parameters other than body and organ weights were calculated using Microsoft Excel software (Microsoft Corporation, Redmond, WA). One-way analysis of variance (ANOVA) was used to test if there was a statistically significant trend in the data (GraphPad Software, Inc., San Diego, CA). If group variances were not significantly different, the ANOVAs were performed with a Dunnett’s post-test to assess differences between the control and brevetoxin-exposure groups. If group variances were significantly different, the Kruskal-Wallis test was used coupled with Dunn’s post-test for comparisons of exposed versus the control groups.

Group mean body weight and organ weight data were tested for statistical significance using Path-Tox software. Bartlett’s test was used to establish the homogeneity of the data. If the data were homogeneous, significance was evaluated using a modified Dunnett’s test. If data were nonhomogeneous, a modified *t*-test was used. For all parameters, the criterion for significance was *p* < 0.05.

## Results

### Exposure atmosphere and estimated breve-toxin 3 respiratory tract deposition.

The characteristics of the exposure atmospheres are summarized in Table 1. Mean total aerosol concentrations for the control chamber, the low-brevetoxin chamber, and high-brevetoxin chamber were 21.6, 5, and 22.6 mg/m^3^, respectively. Mean brevetoxin 3 concentrations, determined by liquid chromatography–mass spectrometry (LC-MS) analysis of aerosol filter extracts for the low- and high-level groups, were 37 and 237 μg/m^3^, respectively. Brevetoxin concentrations obtained and by ELISA were similar to the values obtained by LC-MS. The particle size distribution indicated that the aerosol was highly respirable in rats. Assuming a minute volume of 0.25 L/min and total respiratory tract deposition of approximately 0.2 (for 1 μm particles; Schlesinger 1989), total respiratory tract deposition of brevetoxin 3 in a 2-hr period would be 0.22 and 1.4 μg/day (0.91 and 5.8 μg/kg/day) for the low- and high-exposure groups, respectively. The concentration of ethanol vapor in the control chamber exposure atmosphere was 5.9 g/m^3^. Because control rats displayed no clinical signs of toxicity and no biochemical or histologic change, this ethanol vapor concentration does not appear to be toxic under the conditions of this experiment.

### Body weight gain and clinical signs of toxicity.

**Figure 1:**
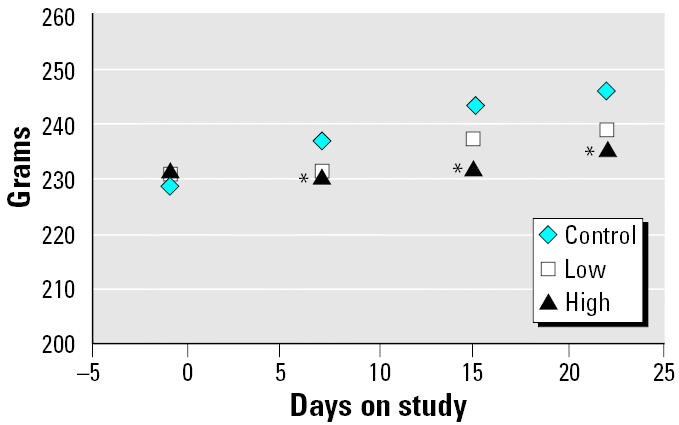
Effect of brevetoxin inhalation on body weight. Values are the mean of ≥ 14 values.
*Statistically different from control, ANOVA *p* < 0.05.

Mean group body weights of rats inhaling the high brevetoxin 3 concentration were significantly below control values 1 week after initiation of exposure with the effect persisting throughout the exposure period (Figure 1). No clinical signs of toxicity were observed at any time.

### Terminal body and organ weights.

Terminal body weights were (mean ± SD, *n* = 6) 251 ± 9.20, 236 ± 9.49, and 227 ± 7.38 for the control, low-, and high-brevetoxin groups, respectively. The terminal body weights for the low- and high-exposure groups were significantly below the control values. Absolute liver weights for low- and high-exposure groups and absolute kidney weight for the high-exposure group were also significantly lower than their respective control values (data not shown). Percent organ to body weight data for liver and kidney indicate that the depression in liver and kidney weights was secondary to overall brevetoxin 3–induced body weight depression.

### Histopathology.

**Figure 2:**
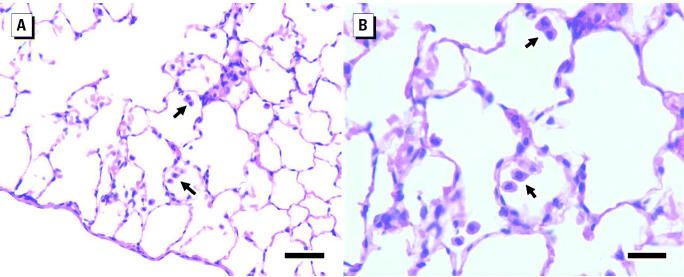
Increased numbers of alveolar macrophages in the alveoli of a rat exposed to high-brevetoxin concentration, a typical presentation. (*A*) Bar = 50 μm. (*B*) Bar = 25 μm. Arrows indicate macrophages.

The only lesion observed in the brevetoxin-exposed rats was alveolar macrophage hyperplasia of minimal severity. The incidences among the control, low-, and high-exposure subgroup A rats were 0 of 6, 3 of 6, and 6 of 6, respectively. The hyperplasia was characterized by a slight increase in the number of alveolar macrophages in the lung and occasional alveoli, where two to four were congregated (Figure 2). Macrophages were also found in the lumens of terminal bronchioles, an atypical location for these cells. The cytoplasm of the macrophages appeared normal and was not vacuolated, enlarged, or pigmented. No evidence of neuronal damage or loss was detected in sections of the hippocampus and cerebellar cortex. Occasional neurons or groups of neurons were crenated and darkly stained. However, there were no tissue changes associated with these cells such as edema, inflammatory cell infiltrates, macrophages, or staining alterations of the neutrophil indicating degeneration of nerve fibers. Therefore, these darkly stained neurons were interpreted as fixation artifacts.

### Clinical pathology.

Brevetoxin inhalation had no significant effect on the total or differential white blood cell counts (Table 2). The numbers of reticulocytes were significantly increased in the high-exposure group, and there were decreasing trends in mean corpuscular volume (*p* < 0.042) and in the mean corpuscular hemoglobin concentration (*p* < 0.051). However, although there were slight decreases in the numbers of red blood cells, hematocrit, and hemoglobin with increasing exposure levels, these trends were not statistically significant.

### Lavage fluid parameters.

The bronchoalveolar lavage fluid obtained from the right lungs of control rats contained (mean ± SD, *n* = 6) 159 ± 47 mIU of LDH activity, 0.41 ± 0.18 mg of total protein, and 4.67 ± 0.93 million total nucleated cells. Macrophages comprised ≥ 98% of the nucleated cell population. Results of the chemilumenescence assays indicated that brevetoxin inhalation did not affect baseline macrophage activity or the response of macrophages to particle (zymosan) stimulation (data not shown).

### Brevetoxin concentration in liver.

The limit of detection of brevetoxin in the ELISA assay is 1 ng/mL for brevetoxin in buffer. Measured concentrations of brevetoxin and/or metabolites in low-exposure-group rats were not significantly different from control values after 5 and 22 days of exposure (Table 3). Brevetoxin concentrations in liver of high-exposure-group rats were significantly increased above background. Brevetoxin concentrations in high-exposure-group livers after 5 and 22 days of exposure were not significantly different, indicating that no accumulation of brevetoxin or its metabolites in liver occurs with repeated exposure. The reason for the relatively high background level in the control liver 11–12 ng/g liver is not known. However, because control animals were exposed in a chamber designated only for control animals, and all animals were housed individually, it is not likely that the background was due to an inadvertent exposure of the controls to brevetoxin.

### Immune responses.

Repeated inhalation of brevetoxin 3 had no marked effect on spleen weights or numbers of lymphoid cells isolated from spleens (Table 4). Numbers of plaque-forming cells were depressed by > 60% in spleen cells after inhalation. The magnitude of suppression did not appear to be exposure concentration dependent. However, the high-exposure group’s large standard deviation makes it difficult to determine this with certainty.

## Discussion

The purpose of this investigation was to evaluate the possible toxic effects associated with repeated inhalation of brevetoxin 3, one of the major brevetoxins produced by *K. brevis*. Because the occurrence and distribution of *K. brevis*–induced red tide has increased over time, there may be increased risk from brevetoxin-induced toxic effects, especially among individuals working and living along beaches affected by red tides. Few data are available on the effects of prolonged exposure to brevetoxins during red tides.

Rats in this study were exposed to a highly respirable, sodium chloride–based aerosol. The aerosol size (0.9–1.3 μm MMAD) is smaller than that measured along red tide–affected beaches (~ 6–12.2 μm MMAD) (Cheng et al. 2005). However, deposition of both the laboratory-generated brevetoxin-containing aerosols in rats and the larger-sized brevetoxin-containing environmental aerosols in humans is expected to occur primarily in the nasopharyngeal and, to a lesser extent, in the tracheobronchial and pulmonary regions (Schlesinger 1989). Regardless, brevetoxins are highly lipophilic and are likely absorbed from throughout the respiratory tract after aerosol deposition. After absorption, systemic distribution is expected to occur rapidly, with the liver expected to be the organ containing a high proportion of the deposited dose (Benson et al. 1999). We found significant concentrations of brevetoxin and/or its metabolites in the liver of rats after 5 and 22 days of exposure to only the high brevetoxin concentration (237 μg/m^3^). The concentration in liver did not increase with continued exposure between 5 and 22 days, suggesting that accumulation in tissue did not occur with repeated exposure.

We have found no evidence of inflammation in any organ, including the respiratory tract, in rats inhaling up to 237 μg/m^3^ for 2 hr/day for up to 22 days, resulting in an estimated deposition of 5.8 μg compound/kg body weight/day. These results are consistent with the lack of inflammatory responses in Sprague-Dawley rats inhaling *K. brevis* extract containing 50 or 200 μg brevetoxin/m^3^ for up to 4 weeks (Benson et al. 2003) or up to 500 μg/m^3^ for 5 days (Benson et al. 2004). The differences in inflammatory responses between the manatees and rats are likely a function of differences in overall brevetoxin exposure; however, there has been no systematic investigation on the relative sensitivities of marine mammals and rats to brevetoxins.

Although minimal macrophage hyperplasia was noted histologically in the lungs of brevetoxin 3–exposed rats, significant increases in macrophage numbers were not found on examination of bronchoalveolar lavage fluid. Activation of macrophages was not evident either morphologically or on examination of chemiluminescence with or without particle stimulation. Alveolar macrophage hyperplasia was not evident in rats inhaling 500 μg/m^3^ brevetoxin (Benson et al. 2004) for 5 days; therefore, it appears that prolonged brevetoxin inhalation may be necessary to induce minimal changes in macrophage numbers in rat lungs.

We found no evidence for neurotoxicity as evidenced by clinical signs or upon histopathologic examination of the rats. Lack of neurotoxicity, especially in the cerebellum, is notable, in light of the fact that brevetoxin localizes in rodent cerebellum after a single injection (Bourdelais et al. 2004) and is cytotoxic in rat cerebellar granular cells *in vitro* (Berman and Murray 1999). However, the relative dose of brevetoxin to brain is expected to be very low (< 1% of the initial body burden) compared with that distributed to carcass (primarily skeletal muscle; ~ 48%), gastrointestinal tract (~ 35%) and liver (~ 10%) based on results obtained after acute administration via the lung (Benson et al. 1999). Slow absorption of brevetoxin that occurs with an inhalation exposure compared with rapid uptake of a bolus dose may allow brevetoxin metabolism to less neurotoxic metabolites to occur, or allow time for uptake into tissue sinks such as skeletal muscle. For example, Poli et al. (1990) have demonstrated decreased neurotoxicity in rats upon slow infusion of brevetoxin 3 by injection compared with administration of a bolus dose.

There was some evidence that brevetoxin inhalation may affect the hematopoietic system. Reticulocyte numbers in blood were significantly increased in the brevetoxin high-dose group; however, there was no significant effect on erythrocyte count, hemoglobin concentration, or hematocrit; and there were no associated histologic changes in bone marrow. Decreasing trends in mean corpuscular hemoglobin volume and corpuscular hemoglobin concentration were consistent with the observed increase in reticulocyte counts. The no-effect level for the increases in reticulocyte counts in rats was 37 μg of brevetoxin/m^3^, a concentration orders of magnitude higher than the nanogram per cubic meter values measured to date along Florida beaches during red tide events (Cheng et al. 2005). Although our hematologic findings with brevetoxin 3 to some extent support the findings of hemolytic anemia in manatees exposed to high brevetoxin concentrations for long periods (Bossart et al. 1998), the implications for humans after exposure are limited.

Observed decreases in blood urea nitrogen and triglyceride levels in serum of high-exposure rats could be associated with reduced caloric intake, suggested by reduced body weights in this dose group for most of the exposure period.

Our findings of suppressed antibody cell production is consistent with our earlier findings in rats inhaling *K. brevis* extract for 4 weeks as well as pure brevetoxin 3 for 5 days (Benson et al. 2003, 2004) and reports of brevetoxin uptake by macrophage and lymphocytes in manatees (Bossart et al. 1998). This finding is probably the most important in relation to brevetoxin inhalation because we have yet to identify a no-effect level in rats. Therefore, it is possible that some immune suppression may occur at exposure concentrations encountered in the environment along red tide affected beaches.

The formation of IgM antibodies in the plaque-forming assay used requires the interaction of macrophages and B- and T-lymphocytes, so the functioning of any or all of these cells may have been affected by brevetoxin exposure (Luster et al. 1988). Although brevetoxins may have affected immune function by several mechanisms, the absence of histopathologic or changes in spleen weight or splenocyte numbers, changes in splenocyte profiles, or responses to mitogens suggest a mechanism independent of overt cytotoxicity. Cathepsin inhibition is one likely mechanism underlying brevetoxin-induced suppression of antibody production because *a*) alveolar macrophages, macrophages within bronchus associated lymphoid tissue, and spleen of brevetoxin-exposed rats stain positively for brevetoxin (Benson et al. 2003, 2004); *b*) brevetoxin 2 is a potent inhibitor of cathepsin L *in vitro* (Sudarsanam et al. 1992); and *c*) cathepsins located within macrophages and B-lymphocytes play an important role in the formation of antigenic determinants essential for both humoral and cell-mediated immune responses (Katunuma et al. 2003; Lewis et al. 1998; Riese and Chapman 2000). One example of an immunotoxicant that inhibits primary antibody production *in vivo* by suppressing splenic cathepsin activity is gallium arsenide (Lewis et al. 1998). No-effect levels for brevetoxin-induced suppression of antibody responses, effects on overall immune competence, and the role of cathepsin inhibition in these processes are the foci of future investigations.

## Conclusions

Inhalation of 37 and 237 μg/m^3^ of brevetoxin 3 in a sodium chloride–based aerosol resulted in some significant reduction in body weight, minimal alveolar macrophage hyperplasia, and some alterations in hematologic parameters. No neurotoxic effects were found. The most significant effect of repeated brevetoxin 3 inhalation was the suppression of antibody production by splenic lymphocytes. This suppression has been seen after repeated inhalation of *K. brevis* extract for 4 weeks and with inhalation of brevetoxin 3 for 1 and 4 weeks in rats. These studies were conducted in young, healthy rats exposed for short periods of time each day, although to higher brevetoxin concentrations than expected to occur in the environment. Potential adverse health effects on the very young or aged laboratory animals remain to be determined. Therefore, much remains to be learned about the long-term health effects of repeated inhalation exposure to brevetoxins. The reader is referred to studies by Fleming et al. (2005b) and Abraham et al. (2005) for discussion of the effects of inhaled brevetoxins on respiratory function in asthmatic humans and in an animal model of allergy, respectively.

## Figures and Tables

**Table 1:** Exposure atmosphere characteristics.

Exposure group	Total aerosol (mg/m^3^*^a^*)	Brevetoxin 3 (μg/m^3^ by mass spectrometry*^b^*)	Brevetoxin (μg/m^3^ by ELISA*^c^*)	Particle size distribution [μm MMAD (σ_g_^)]^
Vehicle control	21.6 ± 4.72	NA^e^	NA	0.92 (2.12)
Low brevetoxin 3	5.06 ± 0.50	37.4 ± 10	44 ± 22	1.33 (2.05)
High brevetoxin 3	22.6 ± 1.45	237 ± 87	216 ± 56	1.12 (2.37)

NA, not applicable.

a Mean ± SD; *n* = 23.

b Mean ± SD; *n* = 10.

c Mean ± SD; *n* = 6–8.

**Table 2:** Effects of brevetoxin inhalation on selected hematology parameters (mean ± SD; *n* = 6).

Exposure group	WBC (×10^3^ cell/μL)	RBC (×10^3^cells/μL)	Hgb (g/dL)	Hct (%)	MCV (fL)	MCH (pg)	Reticulocytes (cells/μL)
Vehicle control	2.75 ± 0.41	8.63 ± 0.34	15.03 ± 0.44	47.18 ± 2.49	54.7 ± 0.88	17.4 ± 1.19	169 ± 13.3
Low brevetoxin 3	3.35 ± 0.76	8.66 ± 0.39	15.12 ± 0.69	46.87 ± 1.76	54.2 ± 0.96	17.5 ± 0.14	194 ± 31.4
High brevetoxin 3	3.27 ± 0.43	8.49 ± 0.19	14.65 ± 0.31	45.47 ± 1.19	53.6 ± 0.69	17.3 ± 0.08	240 ± 13.8 *

Abbreviations: Hct, hematocrit; Hgb, hemoglobin; MCH, mean corpuscular hemoglobin concentration; MCV, mean corpuscular volume; RBC, red blood cell count; WBC, white blood cell count.

* Mean significantly different from control (*p* ≤ 0.05).

**Table 3:** Concentrations of brevetoxin 3 or metabolites in rat liver (mean ± SD; *n* = 3–5).

	5-Day sacrifice		22-Day sacrifice	
Exposure group	ng/g liver	ng/liver	ng/g liver	ng/liver
Vehicle control	12.1 ± 1.28	106 ± 12.0	11.4 ± 0.44	93.0 ± 5.72
Low brevetoxin 3	16.3 ± 3.39	142 ± 29.8	13.7 ± 1.77	106 ± 18.7
High brevetoxin 3	22.7 ± 7.11 *	205 ± 64.9 *	29.9 ± 8.60 *	215 ± 63.2 *

* Mean significantly different from control (*p* ≤ 0.05).

**Table 4:** Effect of brevetoxin inhalation for 22 days on antibody-forming cell response (mean ± SD; *n* = 4–5).

Exposure group	Spleen weight	Lymphocytes × 10^6^	Plaque-forming cells per 10^6^ lymphocytes
Vehicle control	0.51 ± 0.04	114 ± 29	560 ± 164
Low brevetoxin 3	0.49 ± 0.02	126 ± 33	155 ± 87*
High brevetoxin 3	0.48 ± 0.03	124 ± 32	235 ± 227*

* Mean significantly different from control (*p* ≤ 0.05).
